# Comparative Genomics of *Stenotrophomonas maltophilia* and *Stenotrophomonas rhizophila* Revealed Characteristic Features of Both Species

**DOI:** 10.3390/ijms21144922

**Published:** 2020-07-12

**Authors:** Artur Pinski, Joanna Zur, Robert Hasterok, Katarzyna Hupert-Kocurek

**Affiliations:** Institute of Biology, Biotechnology and Environmental Protection, Faculty of Natural Sciences, University of Silesia in Katowice, 28 Jagiellonska Street, 40-032 Katowice, Poland; joanna.zur@us.edu.pl (J.Z.); robert.hasterok@us.edu.pl (R.H.)

**Keywords:** comparative genomics, degradation of xenobiotics, plant-associated bacteria, plant growth promotion, *Stenotrophomonas maltophilia*, *Stenotrophomonas rhizophila*

## Abstract

Although *Stenotrophomonas maltophilia* strains are efficient biocontrol agents, their field applications have raised concerns due to their possible threat to human health. The non-pathogenic *Stenotrophomonas rhizophila* species, which is closely related to *S. maltophilia,* has been proposed as an alternative. However, knowledge regarding the genetics of *S. rhizophila* is limited. Thus, the aim of the study was to define any genetic differences between the species and to characterise their ability to promote the growth of plant hosts as well as to enhance phytoremediation efficiency. We compared 37 strains that belong to both species using the tools of comparative genomics and identified 96 genetic features that are unique to *S. maltophilia* (e.g., chitin-binding protein, mechanosensitive channels of small conductance and KGG repeat-containing stress-induced protein) and 59 that are unique to *S. rhizophila* (e.g., glucosylglycerol-phosphate synthase, cold shock protein with the DUF1294 domain, and pteridine-dependent dioxygenase-like protein). The strains from both species have a high potential for biocontrol, which is mainly related to the production of keratinases (KerSMD and KerSMF), proteinases and chitinases. Plant growth promotion traits are attributed to the biosynthesis of siderophores, spermidine, osmoprotectants such as trehalose and glucosylglycerol, which is unique to *S. rhizophila*. In eight out of 37 analysed strains, the genes that are required to degrade protocatechuate were present. While our results show genetic differences between the two species, they had a similar growth promotion potential. Considering the information above, *S. rhizophila* constitutes a promising alternative for *S. maltophilia* for use in agricultural biotechnology.

## 1. Introduction

*Stenotrophomonas maltophilia* is a ubiquitous bacterium that is commonly found in water, soil, plant-associated habitats, animal tissues and the human body. Its presence has also been detected in extreme environments such as soda lakes, corroded metal surfaces and oil brine [1,2]. This bacterium plays an essential role in the functioning of ecosystems, mainly in the sulphur and nitrogen cycles, the degradation of xenobiotics and plant growth promotion [3]. *S. maltophilia* was extensively applied as a biocontrol or stress-protecting agent for crops and plant growth promotion until the 1980s when it was also reported as being a new hospital pathogen. Since then, *S. maltophilia* has become an emerging global pathogen that is responsible for bacteraemia and respiratory tract infections [4]. Because of the low outer membrane permeability that is characteristic for Gram-negative bacteria and its vast intrinsic resistance to various groups of antibiotics including carbapenems and aminoglycosides as well as its high level of genetic diversity, *S. maltophilia* poses a serious threat to human health and presents a great challenge in the treatment of dieses caused by bacteria [5]. Surprisingly, the *S. maltophilia* strain is also considered to be one of the potential causative agents that was involved in the decline of Persian oak in Iran [6]. The opportunistic human pathogens that dwell in environmental reservoirs are referred to as environmental pathogens [7]. Such behaviour is possible due to the similarities between the mechanisms that are responsible for the colonisation of plant and human organisms. Among them, a few of the features that seem to play an essential role are the ability to form a biofilm, motility, resistance to the osmotic and oxidative stress and the ability to synthesise antimicrobial compounds [5]. 

Because *S. maltophilia* isolates constitute a threat to human health, it is vital to look for other, non-pathogenic bacteria that have a great potential for use in agricultural biotechnology. In 2002, a new *Stenotrophomonas* species called *S. rhizophila,* which is a non-pathogenic, plant-associated habitant, was described by Wolf [1]. To date, the bacteria that belong to this species have been found in associations with various plants, i.e., endemic *Astragalus terraccianoi*, which grows on Mediterranean islands; the metal-tolerant *Biscutella laevigata*; the nickel hyperaccumulator *Noccaea caerulescens*; in the rhizosphere of the important forage grass *Lolium perenne* as well as in bulk soil [8,9,10]. As was shown by Tian et al. [11], the bacteria of this species constitute the core microbiome of tomato roots that are cohabitated with *Pseudomonas*, *Bacillus* and *Rhizobium* genera. Moreover, *S. rhizophila* was identified as being one of the species that colonise decaying alder nodules [12]. Because of its ability to grow at 4 °C and its inability to proliferate at 37 °C, *S. rhizophila* can be distinguished from *S. maltophilia*. Because it cannot grow at 37 °C, this decreases the possibility of human infections and to date, there has been no report on any pathogenic strains of *S. rhizophila* [5,13]. The *S. rhizophila* isolates, in contrast to *S. maltophilia,* are capable of using xylose as a source of carbon and also exhibit a lower osmotic tolerance despite the production of the osmoprotectants such as trehalose and glucosylglycerol [1]. The presence of *ggpS*, a gene encoding glucosylglycerol-phosphate synthase that is required for glucosyl glycerol biosynthesis and the absence of the *smeD* gene encoding part of the multidrug efflux pump, SmeDEF serve as robust genetic markers for the *S. rhizophila* strains [14]. Even though the features that differentiate both species are well defined, the knowledge about *S. rhizophila* is still limited. One attempt to characterise the possible risks for humans was made by comparing the *S. rhizophila* strain DSM14405 with the human-pathogenic *S. maltophilia* K279a and the plant-associated *S. maltophilia* R551-3 using genomics, transcriptomics and physiological assays [4]. Although this research confirmed the non-pathogenic character of the *S. rhizophila* DSM14405 strain, understanding the interspecies genetic variations and the potential for using *S. rhizophila* in agricultural biotechnology remain elusive.

To date, only a few strains of this species have been sequenced. One of them is *S. rhizophila* QL-P4, which is a strain that efficiently degrades polyvinyl alcohol [15]. Extensive abilities of xenobiotic degradation are widespread in the *Stenotrophomonas* genus and many strains of both *S. maltophilia* and *S. rhizophila* were reported as being excellent degraders [16]. For example, *S. maltophilia* KB2 degrades a variety of aromatic compounds such as phenol, catechol, cresols, benzoic acid, 4-hydroxybenzoic acid, protocatechuic acid, vanillic acid and hydroquinone as well as the poly-cyclic pharmaceutical naproxen [17]. Besides their degradation activity, *S. rhizophila* strains were described as promoting plant growth. Among others, *S. rhizophila* MOSEL-tnc3, which has an antifungal activity and promotes canola root growth [18] and *S. rhizophila* ep-17, which exhibited a beneficial influence on soybean plants under salt stress conditions [19]. 

In this study, we characterised the intraspecific genetic variations in the *S. maltophilia* that inhabit diverse niches. Moreover, using comparative genomics tools, we elucidated the genetic differences between *S. maltophilia* and *S. rhizophila*. We also characterised the abilities of these two species to promote the growth of plant hosts as well as to enhance phytoremediation efficiency.

## 2. Results

### 2.1. General Characteristics of S. maltophilia and S. rhizophila Genomes

**Figure 1:**
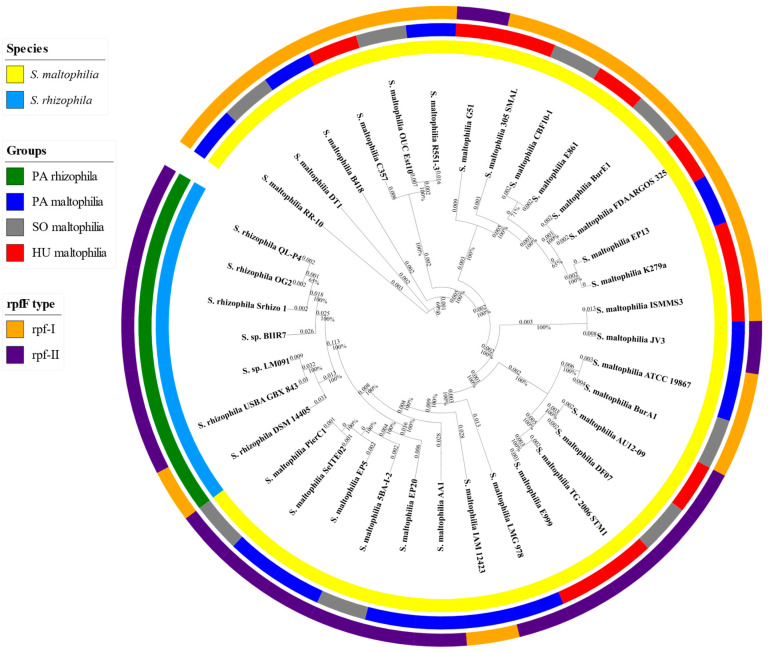
The phylogenetic tree of the 37 analysed *Stenotrophomonas* strains based on their core proteome alignment. The inner track presents the species and the middle track shows the classification of the strains into one of the four specified groups (PA rhizophila, PA maltophilia, SO maltophilia and HU maltophilia). The outer track presents the regulation of pathogenicity factors (*rpf*) type (I or II). The length of a branch is indicated above the node and the support of a branch below the node as a percentage value. The online version of the phylogenetic tree is available at: https://itol.embl.de/tree/94254141149246411589561099#.

**Figure 2:**
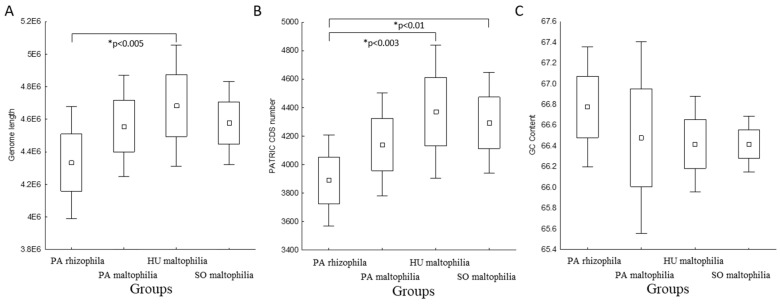
The genomic features of the analysed groups: (**A**) genome length; (**B**) number of CDS; (**C**) GC content. Asterisks indicate the statistically significant differences between the groups that were identified using the Kruskal–Wallis test. Differences in the *p*-value of less than 0.05 were found to be statistically significant and the *p*-value was indicated.

In this study, we analysed 37 genomes of the *S. maltophilia* and S. *rhizophila* species. The genomes were obtained from public databases and were manually classified into one of the four groups: PA rhizophila (plant-associated rhizophila, seven genomes), PA maltophilia (plant-associated maltophilia, 12 genomes), SO maltophilia (soil maltophilia, eight genomes) and HU maltophilia (isolated from human maltophilia, ten genomes). More detailed information about the strains is presented in Table S1. We grouped the 37 genomes into two monophyletic taxa, which correspond to the two *Stenotrophomonas* species, using k-medoids clustering (Figure 1). The differences in the genome length, GC content and the number of coding sequences (CDS) were determined between the grouped strains. Although no statistically significant differences in the GC content were observed, the PA rhizophila group was characterised by a shorter genome length and a lower number of CDS compared to HU maltophilia (Figure 2). SO maltophilia also had a higher number of genes compared to the PA rhizophila group.

**Figure 3:**
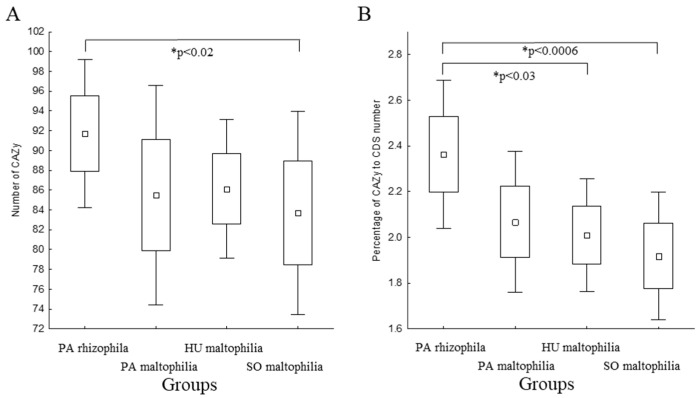
The presence of the genes encoding the carbohydrate active enzymes (CAZymes) in the genomes of the analysed *Stenotrophomonas* strains. (**A**) the number of CAZymes based on the division into the groups; (**B**) the percentage of the CAZymes partition compared to the total number of the CDS based on the division into the groups. Asterisks indicate the statistically significant differences between the groups that were identified using the Kruskal–Wallis test. Differences in the *p*-value of less than 0.05 were found to be statistically significant and the *p*-value was indicated.

Carbohydrate active enzymes (CAZymes) are among the most important enzymes that are involved in metabolising complex carbohydrates. The greater diversity of the CAZymes in plant-related bacteria enables them to utilise the host plant carbohydrates more efficiently, which gives them a competitive advantage [20,21]. Therefore, we sought to identify the CAZymes in the selected strains using the dbCAN2 tool. This approach permitted 204 features to be identified in the pangenome. The number of genes encoding the CAZymes in each genome ranged from 74 to 96. A differential analysis revealed a higher number of CAZymes in the PA rhizophila than in the HU maltophilia (Figure 3A). The difference became more pronounced when the number of CAZymes was considered as a fraction of all of the genes. An analysis that was performed based on this assumption revealed a higher percentage of CAZymes in the PA rhizophila compared to the HU maltophilia and SO maltophilia (Figure 3B). 

**Figure A1:**
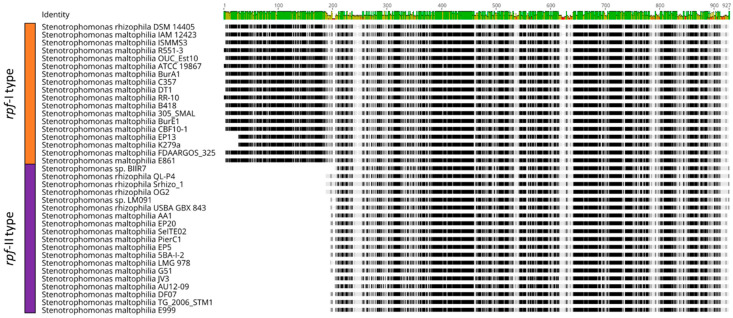
The alignment of the protein sequences of the RpfC from the 37 analysed *Stenotrophomonas* genomes. The *rpf*-I type is characterised by the presence of the N-terminal extension of the RpfC protein, which is not present in the proteins that belong to the *rpf*-II type.

One of the characteristics of *Stenotrophomonas* is the presence of a quorum-sensing system that is based on the diffusible signal factor (DSF) molecule. DSF synthesis is catalysed by the RpfF, which is an enoyl-coenzyme A hydratase that is encoded by the *rpfF* gene that is part of the *rpf* gene cluster. In addition to RpfF, the *rpf* cluster encodes the aconitase RpfA, the fatty acid ligase RpfB, the two-component sensor-effector hybrid system RpfC/RpfG that comprises the sensor kinase RpfC and the cytoplasmic regulatory element RpfG. An analysis of the *rpfC* gene structure led to the identification of two clusters—the *rpf*-I type, which had an extended N-terminal part of the RpfC protein and DSF production and the *rpf*-II type, which produced no DSF [22]. The alignment of the protein-coding sequences of this gene showed the presence of the *rpf*-I type in 18 strains and the *rpf*-II type in 19 strains (Figure A1). The *rpf*-I type was found predominantly in *S. maltophilia* regardless of the occupied niche and only in the *S. rhizophila* DSM14405 strain (Figure 2). 

**Figure 4:**
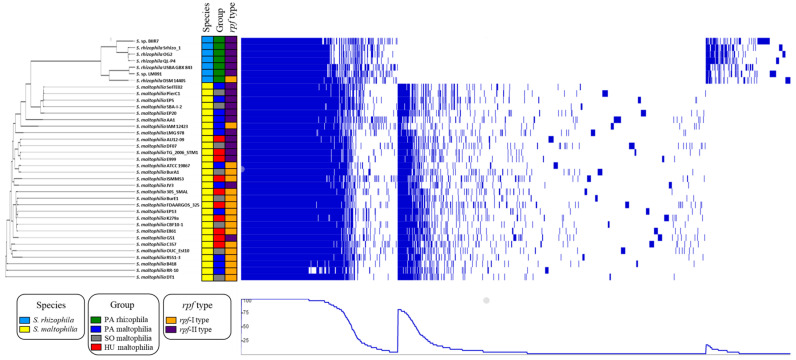
Visualisation of the genomic features of 37 *Stenotrophomonas* strains. The phylogenetic tree is based on the core proteome alignment. The tracks from left to right show the species, group and *rpf* type. The pangenome matrix shows the presence of genes (in blue) in each genome. The raw data that was used for the visualisation in the online tool phandango (https://jameshadfield.github.io/phandango/#/main) are presented in the Supplementary Materials (Table S3, Table S4 and File S1).

**Figure A2:**
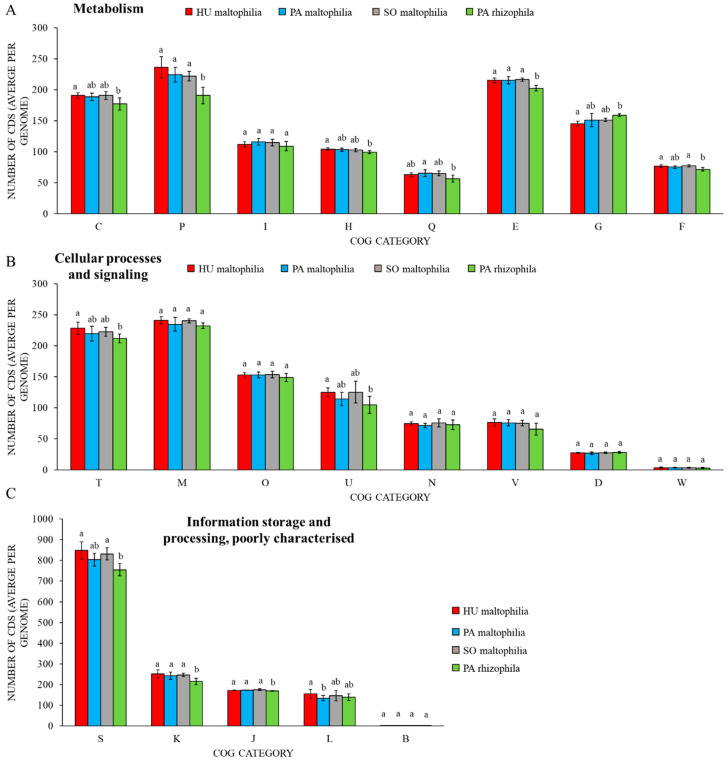
The average COG profiles of the four groups: PA rhizophila (seven genomes), PA maltophilia (12 genomes), SO maltophilia (eight genomes), HU maltophilia (ten genomes). Bar plot showing the average number of CDS per genome per COG category. (**A**) metabolism: COG categories: C, energy production and conversion; P, inorganic ion transport and metabolism; I, lipid transport and metabolism; H, coenzyme transport and metabolism; Q, secondary metabolites biosynthesis, transport and catabolism; E, amino acid transport and metabolism; G, carbohydrate transport and metabolism; F, nucleotide transport and metabolism; (**B**) cellular processes and signalling: T, signal transduction mechanisms; M, cell wall/membrane/envelope biogenesis; O, posttranslational modification, protein turnover, chaperones; U, intracellular trafficking, secretion and vesicular transport; N, cell motility; V, defence mechanisms; D, cell cycle control, cell division, chromosome partitioning; W, extracellular structures; (**C**) information storage and processing, poorly characterised: S, function unknown; K, transcription; J, translation, ribosomal structure and biogenesis; L, replication, recombination and repair; B, chromatin structure and dynamics. Different letters mean significant differences in the Kruskal–Wallis test by ranks and error lines indicate standard errors. Differences with the *p*-value less than 0.05 were considered to be statistically significant. The data that were used for the graphs are presented in Table S7.

The pangenome of the analysed *Stenotrophomonas* strains together with a phylogenetic tree and information about the niche and *rpf* type, were visualised using the phandango tool (Figure 4). Three groups of genes were distinguished: (1) genes that were present in all of the strains and genes that were specific for (2) the *S. maltophilia* and (3) *S. rhizophila* strains. An analysis of the profiles of the COG categories in the four analysed groups is presented in Figure A2 and Table S2.

### 2.2. Characteristic Features of S. maltophilia

We analysed the pangenome matrix using Scoary in order to identify any genetic differences within the *S. maltophilia* species that inhabit diverse niches. The pangenome matrix was obtained through clustering using USEARCH (Table S5) and the protein sequences of the centroids are available in the FASTA file (Supplementary Materials File S2). The analysis of pangenome matrix enables the accurate identification of the genes that were correlated with a specific environment. We compared three *S. maltophilia* groups, but did not observe any statistically significant differences in the gene composition. Therefore, we investigated the genetic differences between *S. maltophilia* and *S. rhizophila* and found them to be substantial. We identified 283 genetic features that were characteristic for *S. maltophilia*, 96 of which were specific for this species (Table S6). The 158 genes were annotated as hypothetical proteins. The remaining features were classified into the COG groups: an unknown function (56), transcription (26), inorganic ion transport and metabolism (21), signal transduction (19), amino acid metabolism and transport (17), intracellular trafficking and secretion (12), cell wall/membrane/envelop biogenesis (9), and others. 

Among the characteristic features, we identified the mechanosensitive channels that protect cells against hypoosmotic shock by sensing changes in the tension of the cytoplasmic membrane and allowing ions and molecules to pass through. Two distinctive classes were revealed: the mechanosensitive channel of small conductance (MscS) and a large conductance mechanosensitive ion channel (MscL). Often, multiple family members were present in a genome, which resulted in different function and expression profiles [23]. The rhizosphere of pea induces the expression of two *mscS* genes in *Rhizobium leguminosarum* [24]. In the analysed genomes of *Stenotrophomonas*, from four to nine of these genes were identified (Table S5). Moreover, one was identified that was unique for the *S. maltophilia* mechanosensitive channel of small conductance (Table S6), which may confer an increased resistance to the hypoosmotic shock that bacteria encounter when colonising the rhizosphere and other hypoosmotic environments. Another feature is that the *ltrA* gene encodes the low temperature requirement protein A, which is essential for the growth of *Listeria monocytogenes* at 4 °C [25]. The homologues of this gene were present in all of the *S. maltophilia* genomes except for the *S. maltophilia* IAM 12423 strain (Table S6). Interestingly, the KGG repeat-containing stress-induced protein was, among others, characteristic for the *S. maltophilia* proteins and some of the strains even had two copies (Table S6). This protein is essential for the stationary-phase resistance to thermal stress and, in particular, acid stress [26]. The inherent resistance of *S. maltophilia* to rifampicin is the result of the rifampicin ADP-ribosyl transferase activity that is encoded by the *arr* gene [27], which is widely distributed in environmental bacteria [28]. Besides the *S. maltophilia* AA1 and *S. maltophilia* EP5 strains, the *arr* gene was absent in the genomes of *S. rhizophila* (Table S5). 

Iron plays an important role in the metabolism of bacteria because its acquisition determines survival and virulence [29]. The genes that are involved in the ferric citrate metabolism were present in both species, though, by far, more of the genes that are responsible for regulating the iron acquisition were present in the genomes of *S. maltophilia*, among which were three *fecR* genes that were absent only in *S. maltophilia* AA1 (Tables S5 and S3). The degradation of chitin, which is a primary component of the fungal cell wall, is one of the most important biocontrol mechanisms. Chitin-binding proteins aid the activity of the chitinases via an increased substrate affinity and enhanced catalytic efficiency [30]. Although chitinase genes were present in the genomes of all of the analysed strains, the genes encoding chitin-binding proteins were only present in the genomes of *S. maltophilia*. Up to three copies of these genes were identified. An analysis using the dbCAN2 tool revealed that chitin-binding proteins belong to the Carbohydrate-Binding Module Family 73. As was shown for *Cellvibrio japonicus*, the Carbohydrate-Binding Module Family 73 protein has a lytic polysaccharide monooxygenase module, which is active toward α- and β-chitin. The chitin-binding domains enhance its activity [31].

The secretion systems are important for interactions with plant hosts and the inactivation of the type III or VI secretion systems in the endophytic strains led to decrease colonisation capabilities [20]. The type II secretion system (T2SS), which is involved in the transport of folded proteins from the periplasm into the extracellular environment, is conserved in most Gram-negative bacteria [32]. In the genomes of *S. maltophilia*, there were additional genes that encode some of the structural proteins of T2SS (referred to as the general secretion pathway proteins D, F, G, H and J) (Table S6). The formation of exopolysaccharide is required by many bacteria for biofilm formation, which helps them to colonise plant and human hosts. The *exoD* gene that encodes the exopolysaccharide synthesis is one of the genes that were characteristic for *S. maltophilia* (Table S6). This gene is essential for exopolysaccharide production and its inactivation in *Rhizobium meliloti* results in an impaired nodule invasion [33].

In the genomes of *S. maltophilia* SmeABC and SmeDEF multidrug efflux pumps play an important role in conferring resistance to various antibiotics [4,34]. The genes encoding SmeDEF multidrug efflux pump were found in genomes of all analysed strains, but genes encoding SmeABC were identified only in a few of *S. maltophilia* strains belonging to all groups (Table S5). Interestingly, nine out of ten HU maltophilia strains featured genes encoding SmeABC pump.

### 2.3. Characteristic Features of S. rhizophila

A comparison of *S. maltophilia* and *S. rhizophila* identified 139 genetic features that were characteristic for *S. rhizophila,* which are presented in Table S7. Among these 139 genes, 59 were exclusively present in all of the analysed *S. rhizophila* strains and were absent in the *S. maltophilia* strains. Of the 139 that were characteristic for *S. rhizophila*, 50 were annotated as being hypothetical proteins. Based on the COG annotation of the remaining genetic features, they were classified as proteins with an unknown function (33), signal transduction (13), transcription (11), cell wall/membrane/envelop biogenesis (9) and others. More detailed information is available in Table S7. 

Among the features that were exclusively present in the *S. rhizophila* strains was the *thuA* gene, which encodes an enzyme that is involved in trehalose utilisation. The *thuA* and *thuB* genes are induced at the surface of the root and in the infection threads. A disruption of one of them leads to impaired root colonisation [35]. Ampomah et al. [36] found that the *thuAB* genes are involved in the utilisation of trehalose and maltitol via the formation of their 3-ketoderivatives. It is still unresolved whether these genes work simultaneously or sequentially. Only the *thuA* gene was found in the *S. rhizophila* strains (Table S7). Thus, the presence of *thuA* may increase that ability of the *S. rhizophila* strains to colonise during the early stages.

Besides DSF, some bacteria are capable of diffusible factor biosynthesis that belong to group of butyrolactones and their biosynthesis is catalysed by a pteridine-dependent dioxygenase-like protein [37]. We identified the gene encoding this enzyme in the *S. rhizophila* strains (Table S7). Cyclic diguanylate (c-di-GMP) serves as a universal secondary messenger that regulates cell differentiation, adhesion, biofilm formation, motility, colonisation of host tissues and virulence, among others. The c-di-GMP is formed via the diguanylate cyclase with the GGDEF domain and is degraded by the phosphodiesterases that contain either an EAL or HD-GYP domain. A major sub-group of the proteins that are involved in c-di-GMP signalling contains both the GGDEF and EAL domains. In some cases, both domains are active, while in others only one of them is or neither is. As was shown on the example of *Xanthomonas*, the GGDEF-EAL domain proteins usually regulate virulence and motility [38]. The importance of the enzymes with both the GGDEF and EAL domains was shown on an *Azoarcus* sp. BH72 mutant. The inactivation of the gene encoding these enzymes resulted in a reduced root colonisation, possibly due to an alteration of the c-di-GMP level. A similar reduction of colonisation efficiency was observed for a mutant with an inactivated GGDEF domain-containing protein [39]. The 16 clusters of these dual activity proteins were found in the *Stenotrophomonas* strain, but only one of them was exclusively present in *S. rhizophila*. Additionally, the gene encoding diguanylate cyclase is also characteristic for *S. rhizophila* and was present in all of the strains except *Stenotrophomonas sp.* BIIR7. However, the characteristics for an *S. maltophilia* gene cluster of diguanylate cyclase was also identified, thereby suggesting great differences in the protein sequences between these two species. 

In bacteria, the cold shock proteins (CSP), which act as an RNA chaperone, are highly expressed during stress acclimation and periods of high metabolic activity. The expression of bacterial CSP in transgenic plants improves their tolerance to abiotic stresses including cold and a water deficit [40]. The gene encoding CSP was found in all of the analysed strains, but only in *S. rhizophila* was there an additional gene encoding CSP with the DUF1294 domain located at the C-end of the protein (Table S7). 

Plants use a variety of defence mechanisms to protect themselves from microorganisms, including the production of reactive oxygen species, nitric oxide and phytoalexins. In order to survive in this challenging environment, plant-associated bacteria must have the ability to quickly adapt to and cope with oxidative stress. Glutathione S-transferase participates in the detoxification of the reactive electrophilic compounds [41]. Many of the genes encoding glutathione S-transferase were found in the *Stenotrophomonas* genomes, but one gene seemed to be characteristic for *S. rhizophila* (Table S7). Generally, the number of glutathione S-transferase in *S. rhizophila* was higher and ranged from 9 to 11, while, in *S. maltophilia*, it ranged from 5 to 9. Similar to the glutathione S-transferase, we identified a catalase that was specific for *S. rhizophila*. Moreover, more catalase-encoding genes were found in *S. rhizophila* (4 to 6) compared to the *S. maltophilia* (2 to 5). Likewise, three different oxidoreductases were identified in all of the *S. rhizophila* strains (Table S5). Interestingly, an aldehyde dehydrogenase that was exclusive for *S. rhizophila* was found to be similar to the moss (*Tortula ruralis*) aldehyde dehydrogenase ALDH21A1. It has been suggested that this dehydrogenase plays a role in the detoxification of the aldehydes that are generated in response to desiccation and salinity stresses [42]. Taken together, this may suggest a higher capacity of the *S. rhizophila* strains to cope with oxidative and salinity stresses.

### 2.4. Genes Encoding the Proteins that Are Involved in Plant Growth Promotion

The plant growth promotion abilities of the bacteria from the *Stenotrophomonas* genus predominantly rely on their biocontrol properties [43]. Fang et al. [44] identified two keratinases, KerSMD and KerSMF, in *S. maltophilia* BBE11-1. KerSMD is characterised by a higher activity of feather degradation, thermostability, substrate specificity and tolerance to surfactants such as SDS and Triton X-100 than KerSMF. Both enzymes were identified in all of the analysed *Stenotrophomonas* genomes (Table S5). An important role for the efficient biocontrol of phytopathogens is also played by the extracellular proteases. Although the genes encoding proteases were identified in most of the genomes of the analysed strains, a higher number of these genes were found in *S. maltophilia (*Table S5). Chitinases and the chitin-binding proteins, which exert a synergistic effect on specific chitinases that increases their activity, also play important roles in the biocontrol processes [45]. An analysis of the *Stenotrophomonas* genomes revealed the presence of two different chitinases in the genomes of all of the analysed strains as well as some additional chitinases, but the chitin-binding proteins were characteristic for *S. maltophilia* (Tables S5 and S3). 

Trehalose is a nonreducing disaccharide that is produced by the various organisms, which protects against different abiotic stresses such as drought, high salinity and temperature extremes. Overproduction of this disaccharide by plant-related microorganisms can increase the tolerance of its host to the abiotic stresses, thereby resulting in efficient plant growth promotion [46]. The genes that are required for trehalose biosynthesis from UDP-glucose and glucose-6-phosphate were present in the genomes of the analysed strains (Table S5). Additionally, in all of the analysed genomes, the gene encoding trehalose-6-phosphate hydrolase was also present. The product of this gene competes with trehalose-6-phosphate phosphatase, which catalyses the biosynthesis of trehalose from alpha,alpha-trehalose-phosphate. In *Escherichia coli*, trehalose-6-phosphate hydrolase under low osmolality conditions degrades alpha,alpha-trehalose-phosphate to glucose and glucose-6-phosphate [47]. Moreover, in a few of the analysed strains, the genes that are required for trehalose synthesis from starch were also present (malto-oligosyltrehalose trehalohydrolase and malto-oligosyltrehalose synthase) as well as trehalase, which catalyses the conversion of trehalose to glucose (Table S5) [48]. The synthesis of an additional osmolyte, glucosylglycerol, which confers salt resistance, is catalysed by the glucosylglycerol-phosphate synthase. The gene encoding this enzyme was exclusively present in the genomes of *S. rhizophila*, together with *ycaD*, which is major facilitator that is essential for the transport of this osmolyte (Table S7) [4]. 

The production and excretion of the polyamines play a role in growth promotion. Notably, spermidine, which is produced by *Bacillus amyloliquefaciens* SQR9, enhances the salt tolerance of plant [49]. In *Bacillus subtilis* OKB105, spermidine was a pivotal plant growth-promoting compound [50]. In all of the analysed genomes, the genes that are required for the biosynthesis of polyamine as well as its export were present (Table S5). 

Siderophores are small peptide molecules that bind ferric ions. Siderophore-producing microbes can prevent or reduce the proliferation of phytopathogens by reducing the available iron and can simultaneously provide iron to the plant [51]. In all of the genomes of both of the analysed *Stenotrophomonas* species, the genes that are required for the biosynthesis of catecholate siderophore were present (Table S5). Further research suggested that it is a novel catecholate siderophore that is distinct from enterobactin [52]. The TonB-dependent enterobactin receptor FepA, which is required to utilise the catecholate siderophore was only present in the *S. maltophilia* genomes (Tables S5 and S3). Another receptor that is involved in iron uptake that is encoded by the outer membrane adhesin-like gene (*fhuA* gene) was present in seven of the 37 analysed strains (Table S5). 

Bacteria that solubilise phosphorus convert insoluble organic and inorganic phosphate into a form that can be accessible for plants. The solubilisation of phosphorus can be the result of producing mineral-dissolving compounds such as the hydroxyl ions, organic acids, protons, siderophores and carbon dioxide [51]. Besides the production of organic acids, bacteria are able to secrete phosphatases and phytases, which mobilise the phosphates from organic compounds [53]. Numerous genes encoding the phytases as well as acid and alkaline phosphatases were present in all of the analysed genomes (Table S5). In the *S. rhizophila* species, with the exception of *S. rhizophila* DSM14405, an additional gene encoding acid phosphatase was present (Tables S5 and S4). Similar to the acid phosphatases, alkaline phosphatase participates in mobilising phosphorus via the hydrolysis of organic phosphoesters [53]. In all of analysed genomes, we identified an alkaline phosphatase, which belongs to the ALP-like super family. In *S. maltophilia* (except for *S. maltophilia* IAM 12423 and *S. maltophilia* AA1) an additional alkaline phosphatase that belongs to the PhoD super family, was present. Although the *pstABC* genes that encode the inorganic phosphorus transporter were present in all of the analysed genomes, there was an additional operon coding for this transporter in the *S. maltophilia* ISMMS3 (Table S5). 

### 2.5. Degradation of the Xenobiotics

Among the analysed strains, we identified several genes encoding the degradative enzymes. The protocatechuate 3,4-dioxygenase alpha and beta chain (EC 1.13.11.3) were found in eight of the analysed strains of the PA rhizophila, PA maltophilia and SO maltophilia groups (*Stenotrophomonas* sp. LM091, *S. rhizophila* DSM 14405, *S. maltophilia* ATCC 19867, *S. maltophilia* SeITE02, *S. maltophilia* AA1, *S. maltophilia* EP5, *S. maltophilia* 5BA-I-2 and *S. maltophilia* PierC1). Simultaneously, genes for the degradation of protocatechuate were absent in all the strains from the HU maltophilia group (Table S5). Additional enzymes that are involved in the beta-ketoadipate metabolic pathway were identified in eight of the analysed strains and included 3-carboxy-*cis*,*cis*-muconate cycloisomerase (EC 5.5.1.2, synonym beta-carboxymuconate lactonizing enzyme); beta-ketoadipate enol-lactone hydrolase (EC 3.1.1.24), which is engaged in metabolising catechol to beta-ketoadipate, in the benzoate degradation and *ortho* cleavage in the 4-methylcatechol degradation. Moreover, some enzymes, i.e., acetyl-CoA acetyltransferase (EC 2.3.1.9), 3-oxoadipyl-CoA thiolase (EC 2.3.1.174), the 3-oxoadipate CoA-transferase subunit A (EC 2.8.3.6) and the 3-oxoadipate CoA-transferase subunit B (EC 2.8.3.6), which are responsible for incorporating any by-products into the central metabolism were found. For seventeen of the analysed strains, the NAD-dependent benzaldehyde dehydrogenase II-like that has an oxidoreductase activity, which acts on the aldehyde or oxo group of donors, was also found (Table S5).

## 3. Discussion

In this study, which was based on the classification of 37 strains that belong to the *S. maltophilia* and *S. rhizophila* species and the comparative genomics approach, we attempted to prove that, because of its genomic features, *S. rhizophila* can be a promising alternative for the *S. maltophilia* strains. A high-quality phylogenetic tree that was based on the core proteome improved the initial classification of the two strains, *Stenotrophomonas* sp. BIIR7 and *Stenotrophomonas* sp. LM091, which were determined to belong to the *S. rhizophila* species (Figure 1). Based on the phylogenetic tree, both species, *S. rhizophila* and *S. maltophilia,* can be distinguished as was further proven using k-medoids clustering. In the case of the *S. maltophilia* strains, their phylogenetic placement and assignment to one of the groups did not correspond. This observation aligns well with previous findings by Youenou et al. [54] in which strains from the natural environment and those of a clinical origin were compared. The authors found that strain’s phylogeny did not match their origin or antibiotic resistance profiles. Other research showed that the ability of *S. maltophilia* to adapt to lung cystic fibrosis is associated with its consistent genotypic and phenotypic heterogeneity, even though the correlation between genotype and phenotype was poor [55]. Moreover, Steinmann et al. [56] showed that biofilm formation and proteolytic activity were unrelated to the phylogenetic placement or the isolation source. The genomic classification correlated well with the reactivity of the strains against the *S. maltophilia* K279a O-specific antibody as well as partially with the lipopolysaccharide profiles. Notably, the *ggpS* gene that encodes glucosylglycerol-phosphate synthase was found among features unique for *S. rhizophila* (Table S7) proving to be suitable genetic marker for this species [14]. However, contrary to the previous findings, genes encoding the multidrug efflux pump SmeDEF were found to be present in all genomes of both species, *S. rhizophila* and *S. maltophilia* (Table S5). Previously, it was believed that the *smeD* gene was unique for *S. maltophilia* differencing it from *S. rhizophila* and serving as a genetic marker. The primers designed for amplification of the partial sequence of the *smeD* were shown to be specific for *S. maltophilia* [14], but, as we revealed, this is not due to the lack of *smeD* in the genomes of *S. rhizophila* but because of differences in the *smeD* gene sequences. Notably, the SmeDEF pump was found to participate in endophytic colonisation of plant roots [57]. Further analysis of the genomic features showed that genome length and the number of CDS as predicted by the PATRIC of PA rhizophila were smaller than those of HU maltophilia (Figure 2). In bacteria, genome size and complexity are frequently well correlated; a larger genome encodes more genes, which facilitates a more diverse metabolomic complexity and ecological versatility [58]. An analysis of the CAZymes revealed that they were present in a higher number in PA rhizophila than in HU maltophilia (Figure 3). This signifies the importance of CAZyme enrichment for plant-bacteria interactions. Additionally, an analysis of the COG profile showed that there was a statistically significant higher average number of genes for the genome that had been classified to the G category (carbohydrate transport and metabolism) in PA rhizophila than HU maltophilia (Figure A2, Table S2). The mutants of *Pseudomonas simiae* WCS417r that were unable to utilise galactose, galacturonate, glucose, inosine or 2-deoxyribose showed reduced colonisation fitness [59]. Moreover, there was a higher expression of the CAZymes in the rhizosphere compared to the rhizoplane in the plant-colonising *Cellvibrio* sp. Bin79 [60]. 

The presence of the *rpf* type did not correspond to its phylogenetic placement, group or species (Figure 1). Similar observations were made by Huedo et al. [22], who revealed that among 78 *S. maltophilia* clinical isolates, the *rpf*-I type was found in 47 strains, while the *rpf*-II type was present in the remaining 31 strains. The DSF molecules participate in biofilm formation, swarming motility and virulence, among others. Even though bacteria with the *rpf*-II type are unable to produce DSF, their biosynthesis is derepressed when DSF is detected, which makes them a “social cheater” [61]. The importance of DSF was demonstrated by inoculating oil rape seeds with the wild type of *S. maltophilia* R551-3 model strain and its mutants, which were unable to produce DSF. These mutants were impaired in their colonisation ability and growth promotion, and were additionally defective in the formation of cell aggregates in the rhizosphere [62]. DSF also regulates the expression of the genes that are linked to plant growth promotion such as spermidine synthase and the spermidine export protein. In the *rpfF* mutant of *S. maltophilia* R551-3, the expression of these genes was significantly downregulated [62]. Although of *S. rhizophila* strains only DSM14405 has the *rpf*-I type, *S. rhizophila* strains are considered to be efficient plant colonisers [19,63]. This, however, could be attributed to a different regulation system than the one in *S. maltophilia* such as the diffusible factor that regulates EPS and xanthomonadin biosynthesis in the bacteria from *Xanthomonas* species. The diffusible factor and DSF interact with each other and play an important role in regulating virulence [64]. To date, the production of diffusible factors by *S. rhizophila* was not observed under laboratory conditions, but the gene encoding necessary for their biosynthesis pteridine-dependent deoxygenase-like protein was found in the genomes of *S. rhizophila* (Table S7). It is feasible that diffusible factors may also play an important role in regulating the biofilm formation and colonisation of its host plant in *S. rhizophila*.

Analysis of the genes that were present in the *S. maltophilia* strains considering the isolation source showed no differences between the groups. A similar observation was made by Steinmann et al. [56]; no specific evolutionary branches of *S. maltophilia* were detected. This observation is in agreement with the proposed mechanism of the evolution of *S. maltophilia*, i.e., its adaptation to different micro niches is based on a strain-specific acquisition of genes [65]. It is worth mentioning that *S. maltophilia* exhibits a high natural capacity for DNA uptake, a high rate of genomic rearrangements and hypermutator activity, which contribute to its high intra-specific heterogeneity and genomic plasticity [5]. However, the comparative genomics of *S. maltophilia* and *S. rhizophila* strains revealed differences in the gene composition with both strains having unique genetic features (Tables S6 and S7). During the analyses, we identified 96 genetic features that were unique for *S. maltophilia* and 59 for *S. rhizophila*. This number is much lower than was previously obtained when *S. maltophilia* R551-3 and *S. maltophilia* K279a were compared with *S. rhizophila* DSM14405, which indicated that 762 genes were absent in both of the *S. maltophilia* strains and hence were unique to *S. rhizophila* DSM14405. The difference in the number of obtained unique genetic features was mainly due to the inclusion of seven *S. rhizophila* and 30 *S. maltophilia* strains which enabled a much more precise characterisation of the unique genetic features of these species (Table S1). It is also possible that sequencing and analysing the new strains that belong to these species will further lower the number of their unique characteristic genetic features.

The extensive biocontrol properties of the *Stenotrophomonas* species are primarily related to the production of keratinases, extracellular proteases and chitinases [43], which were identified in all of the analysed strains (Tables S5 and S6). KerSMF keratinase was found to exhibit a biocidal activity on the nematodes, *Caenorhabditis elegans* and *Panagrellus* spp. [66], which makes it an efficient biocontrol agent for plant-parasitic nematodes [67]. As was shown by Dunne et al. [68], the mutant of *S. maltophilia* W81 that was unable to produce one of the extracellular proteases failed to efficiently control the pathogenic fungus *Pythium ultimum*. Another *S. maltophilia* G2 strain was more active against the free-living nematode *Panagrellus redivivus* and the plant-parasitic nematode *Bursaphelenchus xylophilus* due to its production of extracellular protease [69]. A mutant of *S. maltophilia* 34S1 in the chitinase gene was less able to control the root-infecting fungus *Magnaporthe poae* [70]. Moreover, the chitinase from *S. maltophilia* C3 was shown to act as a potent biocontrol agent that effectively inhibited the germination of the conidia of *Bipolaris sorokiniana* [71]. The chitinase from the rhizosphere strain of *S. maltophilia* MUJ significantly inhibited the growth of the fungal phytopathogens that belong to the genera *Fusarium*, *Rhizoctonia* and *Alternaria* [72]. Recently, a characterised chitinase from *S. rhizophila* G22 was shown to efficiently degrade colloidal chitin [73]. In terms of the biocontrol properties, the ability of the *S. maltophilia* may be higher than *S. rhizolphila* due to the presence of additional proteases and chitin-binding proteins, which were absent in *S. rhizophila* (Tables S5 and S3). However, the non-pathogenic character of the *S. rhizophila* attributable to its inability to proliferate at 37 °C does not raise any concerns in field applications as biocontrol agents [5,13].

The strains from *S. maltophilia* and *S. rhizophila* had a similar genetic potential for promoting the growth of the plant host (Table S5). However, only the bacteria that belong to the *S. rhizophila* species can synthesise the additional osmolyte, glucosylglycerol, which may promote the growth of its host under stress conditions [63]. Additionally, the *fhuA* gene was identified in the genomes of seven strains that belong to different groups. Although the presence of this gene was previously linked with plant-related species [4,74], our analysis showed that this gene can also be found in the soil- and human-related strains (Table S5). Interestingly, the gene encoding CSP with DUF1294 domain was among the features that were specific to *S. rhizophila*. Its presence may be related to an increased tolerance to environmental stresses and may also contribute to plant growth promotion. The gene encoding this protein was also found in strains from the *Xanthomonas* genus to which numerous phytopathogens belong through a BLAST search [75]. The *Stenotrophomonas* strains can also contribute to an increase in the phytoremediation efficiency because of their ability to degrade xenobiotics [76]. Among the analysed *S. rhizophila* strains, only *Stenotrophomonas* sp. LM091 and *S. rhizophila* DSM 14405 were found to have the genes that are required to degrade protocatechuate. However, other strains that were not sequenced had a high xenobiotic degradation ability, e.g., of those that had been isolated from oil-contaminated soil, the *S. rhizophila* PM-1 strain degraded the aliphatic and aromatic hydrocarbons [77] and the *S. rhizophila* strain that had been isolated from bird’s nests degraded feathers due to the production of the keratinases [78]. *S. rhizophila* strains were also recovered from microbial consortia, which are rich in lignocellulosic compounds, that had been designed to mineralise a plant biomass. The ability to degrade xenobiotics permits the use of *Stenotrophomonas* spp. in the bioremediation and phytoremediation processes [16,17,79].

## 4. Materials and Method

### 4.1. Genome Collection

A total of 37 genomes of the bacteria that belong to the *Stenotrophomonas* genus were retrieved from the IMG and PATRIC databases. To exclude redundant strains a whole genomic average nucleotide identity was computed using the JSpeciesWS Online Service and one of the pair of genomes that had identity higher than 99.995% was excluded [80]. Only the genomes of strains that have a known isolation source were considered and based on this information, the genomes were classified as: plant-associated maltophilia (PA maltophilia), maltophilia that are isolated from humans (HU maltophilia) or from soil (SO maltophilia). The fourth group consisted of strains that were classified as plant-associated rhizophila (PA rhizophila) based on their phylogenetic placement and the presence of the gene encoding glucosylglycerol-phosphate synthase, which is one of the most important defining characteristics of the *S. rhizophila* species. All of the strains from the *S. rhizophila* species were considered to be plant-associated based on characteristics of this species regardless of their isolation source [1]. *S. maltophilia* strains were classified as plant-associated when they were isolated from the rhizosphere or internal tissue of plants. 

### 4.2. Phylogenetic Analysis

In order to generate a phylogenetic tree of the selected genomes, the core proteomes of 37 strains were extracted and aligned using M1CR0B1AL1Z3R using default settings with the minimal identity for proteins to be 80% and with the minimal value of the orthologues to be considered a core set to be 100% [81]. Poorly aligned regions were removed using Gblocks (version 0.91b) [82], which yielded 441 644 amino acids for the 37 genomes. A maximum-likelihood phylogenetic tree was created using IQ-TREE Galaxy (version 1.5.5.3) with the default settings and a 1000 bootstrap resampling value [83,84]. The phylogenetic tree that was created was visualised using the iTOL (version 5) web-based tool [85]. In order to determine the number of taxonomic groups among the analysed strains, the phylogenetic tree was transformed into a distance matrix using the cophenetic function in the R (version 3.5.0) package ape (version 5.2) (https://cran.r-project.org/web/packages/ape/index.html). The groups were determined using k-medoids clustering implemented in the partitioning around the medoids function in the R package fpc (version 2.1-11.1) (https://cran.r-project.org/web/packages/fpc/index.html).

### 4.3. Comparative Genomics and Bioinformatic Analysis

Information about the genome size, GC content and the number of CDS were retrieved from the IMG and PATRIC databases and compared among the four niche groups using the Kruskal–Wallis test. For the clusters of orthologous genes (COG) enrichment analysis, the protein sequences of the genes from all of the analysed strains were mapped to one of the 25 COG categories using eggNOG-mapper (version 4.5.1) [86]. Any gene that was assigned to multiple COG categories was counted as being present in each of the categories. The genes were clustered using UCLUST by searching the proteins that had an identity and coverage higher than 50% compared to the target. UCLUST was used with command: usearch10.0.240_win32.exe -cluster_fast <input_file.fasta> -id 0.5 -target_cov 0.5 -uc <output_file.uc> -centroids <output_file.fasta>. The pangenome matrix from UCLUST was used to identify the niche-specific genes and the genes that differentiated the *S. maltophilia* and *S. rhizophila* species. The pangenome matrix together with the information about the assignment to the one of the niche groups was submitted to the Scoary algorithm that was running the command: python scoary.py -t <niche_group.csv> -g <pangenome_matrix.csv> [87]. A gene cluster was considered to be statistically significant only if the p-value and Bonferroni *p*-value were lower than 0.05. The pangenomes of the 37 strains were visualised using the phandango web-based tool using a phylogenetic tree, which included metadata containing data about the species, its niche group, the *rpf* type and the pangenome matrix that had been generated by UCLUST. The carbohydrate-active enzyme (CAZy) was annotated using the dbCAN2 web server [21], which integrates three different tools (a HMMER search against the HMM database, a DIAMOND search against the CAZy database and a Hotpep search against the conserved CAZyme short peptide database) for an automatic carbohydrate-active enzyme and the gene was considered to encode the CAZymes if two of the tools were positive.

### 4.4. Statistical Analysis 

Data were analysed using Excel 2019 (Microsoft) and Statistica 13.3 (TIBCO Software Inc., Palo Alto, CA, USA). To test the normality of the data distribution the Shapiro–Wilk (*p* < 0.05) test was used. The statistical significance (*p* < 0.05) of any differences were analysed using the Kruskal–Wallis test. 

## 5. Conclusions

In our work, we revealed significant genetic differences between the strains that belong to *S. maltophilia* and *S. rhizophila* and characterised the unique genes that were found in the genomes of the strains of each species. We showed that there are no characteristic genes in *S. maltophilia* related to the different source of isolation, i.e., plant-associated, soil or human. Our results indicated that both species had a similar potential in the ability to promote the growth of the plant host by producing keratinases, proteinases, chitinases, siderophores, spermidine and osmoprotectants such as trehalose and glucosylglycerol, which is specific for *S. rhizophila*. Some of the *S. rhizophila* strains might also be useful for enhancing the phytodegradation efficiency because of the presence of the genes that are required to degrade aromatic compounds. Overall, considering the non-pathogenic character of *S. rhizophila*, the strains of this species constitute a promising alternative for *S. maltophilia* in agricultural biotechnology. Additionally, our results extend the knowledge about the genetic diversity of the *S. maltophilia* and *S. rhizophila* strains.

## Supplementary Materials

Supplementary materials can be found at https://www.mdpi.com/1422-0067/21/14/4922/s1. File S1: The phylogenetic tree of the analysed *S. maltophilia* and *S. rhizophila* strains in the Newick format with the length of the branches and bootstrap values. File S2: The protein sequences of the centroids in the FASTA format that were obtained through clustering with USEARCH. Table S1: The general characteristics of the 37 analysed strains of *S. maltophilia* and *S. rhizophila*. Table S2: The COG distribution in each genome of the 37 analysed strains of *S. maltophilia* and *S. rhizophila.* Table S3: The pangenome matrix of presence/absence of a gene that was used for the visualisation in the online tool phandango (https://jameshadfield.github.io/phandango/#/main). Table S4: The general characteristics of 37 analysed *S. maltophilia* and *S. rhizophila* strains that were used for the visualisation in the online tool phandango (https://jameshadfield.github.io/phandango/#/main). Table S5: The pangenome matrix of the presence/absence of a gene that was obtained through clustering with USEARCH. Table S6: The list of the 283 genetic features that were identified that were characteristic for *S. maltophilia*. Table S7: The list of the 139 genetic features that were identified that were characteristic for *S. rhizophila*.

## Abbreviations

CAZy	carbohydrate-active enzyme
c-di-GMP	cyclic diguanylate
CDS	coding sequence
COG	cluster of orthologous genes
CSP	cold shock protein
DSF	diffusible signal factor
HU maltophilia	maltophilia isolated from human
PA maltophilia	plant-associated maltophilia
PA rhizophila	plant-associated rhizophila
*rpf*	regulation of pathogenicity factors
SO maltophilia	maltophilia isolated from soil
T2SS	type II secretion system
